# Scoping review of patient- and family-oriented outcomes and measures for chronic pediatric disease

**DOI:** 10.1186/s12887-015-0323-x

**Published:** 2015-02-13

**Authors:** Sara D Khangura, Maria D Karaceper, Yannis Trakadis, John J Mitchell, Pranesh Chakraborty, Kylie Tingley, Doug Coyle, Scott D Grosse, Jonathan B Kronick, Anne-Marie Laberge, Julian Little, Chitra Prasad, Lindsey Sikora, Komudi Siriwardena, Rebecca Sparkes, Kathy N Speechley, Sylvia Stockler, Brenda J Wilson, Kumanan Wilson, Reem Zayed, Beth K Potter, on behalf of the Canadian Inherited Metabolic Diseases Research Network (CIMDRN)

**Affiliations:** University of Ottawa, 451 Smyth Road, Ottawa, ON Canada; Montreal Children’s Hospital, McGill University Health Centre, 2300 Tupper Street, Montreal, QC Canada; Newborn Screening Ontario, Children’s Hospital of Eastern Ontario, 415 Smyth Road, Ottawa, ON Canada; National Center on Birth Defects and Developmental Disabilities, Centers for Disease Control and Prevention, 1600 Clifton Road, Atlanta, GA USA; University of Toronto, 27 King’s College Circle, Toronto, ON Canada; Hospital for Sick Children, 555 University Avenue, Toronto, ON Canada; Centre Hospitalier Universitaire Sainte-Justine, 3175 Chemin de la Côte-Sainte-Catherine, Montreal, QC Canada; Western University, 1151 Richmond Street, London, ON Canada; Alberta Children’s Hospital, 2888 Shaganappi Trail NW, Calgary, AB Canada; British Columbia Children’s Hospital, 4480 Oak Street, Vancouver, BC Canada; Ottawa Hospital Research Institute, 725 Parkdale Avenue, Ottawa, ON Canada

**Keywords:** Patient-centered outcomes research, Patient-centered care, Outcomes research, Outcome measures, Metabolism, Inborn errors, Assessment, Patient outcomes, Rare diseases, Family-centered care

## Abstract

**Background:**

Improvements in health care for children with chronic diseases must be informed by research that emphasizes outcomes of importance to patients and families. To support a program of research in the field of rare inborn errors of metabolism (IEM), we conducted a broad scoping review of primary studies that: (i) focused on chronic pediatric diseases similar to IEM in etiology or manifestations and in complexity of management; (ii) reported patient- and/or family-oriented outcomes; and (iii) measured these outcomes using self-administered tools.

**Methods:**

We developed a comprehensive review protocol and implemented an electronic search strategy to identify relevant citations in Medline, EMBASE, DARE and Cochrane. Two reviewers applied pre-specified criteria to titles/abstracts using a liberal accelerated approach. Articles eligible for full-text review were screened by two independent reviewers with discrepancies resolved by consensus. One researcher abstracted data on study characteristics, patient- and family-oriented outcomes, and self-administered measures. Data were validated by a second researcher.

**Results:**

4,118 citations were screened with 304 articles included. Across all included reports, the most-represented diseases were diabetes (35%), cerebral palsy (23%) and epilepsy (18%). We identified 43 unique patient- and family-oriented outcomes from among five emergent domains, with mental health outcomes appearing most frequently. The studies reported the use of 405 independent self-administered measures of these outcomes.

**Conclusions:**

Patient- and family-oriented research investigating chronic pediatric diseases emphasizes mental health and appears to be relatively well-developed in the diabetes literature. Future research can build on this foundation while identifying additional outcomes that are priorities for patients and families.

**Electronic supplementary material:**

The online version of this article (doi:10.1186/s12887-015-0323-x) contains supplementary material, which is available to authorized users.

## Background

Rare pediatric diseases pose unique challenges for the planning and provision of patient-centred health care [1-3]. These challenges arise from the chronicity and complexity of these diseases, combined with small numbers of patients available for empirical research to investigate patient-and family-oriented outcomes [4]. Generating the evidence to fill these knowledge gaps is challenging [5] as outcomes for children are often proxy-reported [6], affect caregivers as well as patients [7,8], and change over time as adolescents transition from pediatric to adult care [9].

Despite these challenges, incorporating outcomes that align with the priorities of patients and their families is increasingly recognized as imperative in evaluative health research [10-13]. This reflects a growing body of literature supporting patient-centred health care [14,15], and related concepts including patient-informed care [16], shared decision-making [17,18], and personalized health care [19,20]. These trends represent an emerging consensus that the perspectives of patients and their families are critical to evaluating health interventions in order to effectively inform improvements in health care [21-23].

As part of a larger program of research designed to advance health outcomes and interventions for children with rare inborn errors of metabolism (IEM) [24], we conducted a broad scoping review of patient- and family-oriented outcomes and self-administered measures of these outcomes for chronic pediatric diseases with features relevant to IEM. Our review addressed the following questions:

1. Which patient- and family-oriented outcomes have been measured in studies of chronic pediatric diseases relevant to IEM?
2. Which self-administered measures have been used to measure the outcomes identified in 1)?

## Methods

Because our questions were broad, we adopted a tailored scoping review approach which is reported in detail elsewhere [25]. Briefly, we established an expert working group to develop a structured review protocol and execute the search and synthesis of reports of relevant studies. The group included those with clinical expertise in managing IEM, an understanding of patient-reported outcomes research in pediatrics, and experience with knowledge synthesis methods. Because there are few studies describing patient/family-oriented outcomes specific to patients with IEM [26], we considered a broader range of diseases with clinical similarities to IEM. Specifically, we identified hallmark characteristics of IEM: (i) etiology and/or manifestation (genetic, metabolic, and/or neurologic); (ii) chronicity (requiring long-term management); (iii) nature/complexity of care (requiring specialist pediatric care involving medical, surgical or nutritional intervention); and (iv) rarity. We used these characteristics to define our eligibility criteria with the exception of disease rarity, as we did not wish to pre-suppose differences in outcomes relevant to rare versus common diseases, i.e., restricting the review to rare diseases would potentially have been limiting in the context of our objectives.

Eligible outcomes were patient- and/or family-oriented, defined using the approach developed by the authors of the Strength of Recommendation Taxonomy Framework [27], a scale developed for ascertaining the extent to which evidence is patient-oriented. Eligible outcome measures were self-administered, to identify those that can be completed without a researcher being present and therefore of broadest potential utility. We operationalized these features, in combination with limitations on report/study characteristics intended to narrow the search yield to sources most relevant to our research objectives and questions, as inclusion criteria using the patient, intervention(s), comparator(s), outcome(s), study design (PICOS) framework [28] (Table 1).

**Table 1:** **PICOS for scoping review of patient- and family-oriented outcomes, measures for children with chronic diseases**

**Patients**	Children and/or adolescents (i.e., 0-18 yrs) with a chronic disease for which etiology/manifestation(s) are genetic, metabolic or neurologic, and which necessitates specialist pediatric care involving medical, surgical or nutritional intervention, and/or; the families/caregivers of these children and/or adolescents.
**Intervention**	Not applicable
**Comparator**	Not applicable
**Outcome**	Patient- and family-oriented (as defined by the SORT framework), and; measured using self-administered instrument(s)
**Study characteristics**	Peer-reviewed, English-language, full journal articles describing primary studies that included ≥5 eligible patients, published 2002-2012

A search strategy was developed iteratively to identify relevant studies while yielding a feasible number of citations. For example, we searched diseases of interest using Medical Subject Headings (MeSH) only, while we combined text word searches with MeSH to identify relevant outcomes. Likewise, while both English- and French-language articles were retrieved with the electronic search strategy, we reviewed only English-language articles. The final search strategy for Medline is available in the Additional file 1.

We screened the returned titles and abstracts with the pre-specified criteria using a liberal-accelerated approach [29] i.e., a first independent reviewer screened all citations and a second independent reviewer screened all titles and abstracts excluded by the first. From citations eligible for full-text screening, a 20% random sample (264 titles) was isolated for a pilot of the full-text screening and data abstraction approaches. Two independent reviewers applied the pre-specified criteria to these, resolving discrepancies using consensus and involving a third-party arbiter when necessary. Data abstraction was completed for 56 eligible articles from the pilot, allowing for assessment of the process and ascertainment of the extent to which saturation of outcomes and measures had been achieved (see Additional file 1). This pilot work also informed the identification of domains, which were broad categories describing groups of outcomes, supported by a leading source in health measurement [30] and corroborated against the domains described by the Patient Reported Outcomes Measurement Information System Pediatric Self- and Proxy-Reported Health Framework [13]. Following the pilot, we applied the same screening and data abstraction strategy to the remaining citations. Data on study characteristics, patient- and family-oriented outcomes, and their self-administered measures were abstracted for all included articles using a standardized form.

We developed an evolving glossary of outcomes to guide their categorization. Outcome measures were abstracted as reported by study authors i.e., interpretation regarding naming conventions used by authors was withheld. To support these efforts at mitigating bias, data abstraction for all included studies was carried out by one independent researcher and verified by a second. Data were tallied and summarized descriptively.

Because the data were drawn from published literature, the study was not subject to ethics review. And while we conducted a scoping review rather than a systematic review, the Preferred Reporting Items for Systematic Reviews and Meta-Analyses (PRISMA) statement [31] was used to inform preparation of this report.

## Results

### Search and Screening

**Figure 1:**
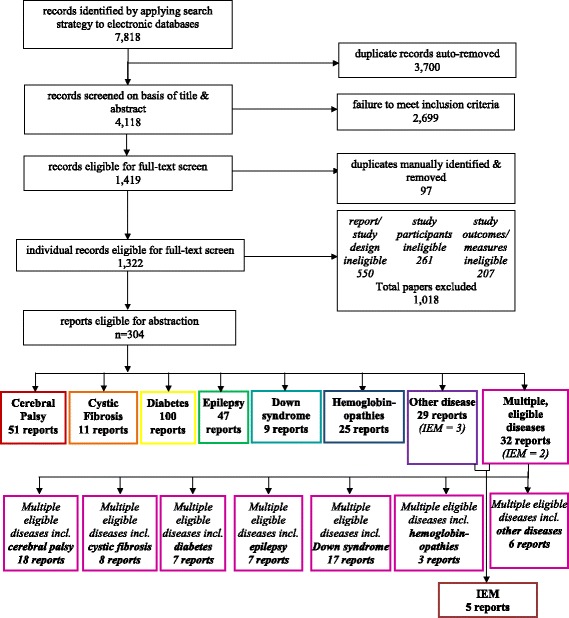
**PRISMA diagram for scoping review of patient- & family-oriented outcomes, measures for chronic, pediatric disease.**

Of 4,118 original citations identified, a total of 304 eligible articles were eligible for inclusion as follows (Figure 1):

Of the 1,322 citations reviewed using full-text, 1,018 were excluded; more than one-third of these (34%) were abstracts and/or non-peer reviewed sources. Another large proportion (20%) described the use of interviewer- and/or clinician-administered outcome measures. A third, considerable proportion (15%) did not report on the use of measures specifically within a pediatric population. Of the 1,322 citations screened at the full-text phase, 55 (4%) required arbitration regarding inclusion, mainly due to lack of clarity in reporting the variables of interest.

### Report characteristics

Of the 304 included articles, eight major categories of disease(s) were identified: cerebral palsy, cystic fibrosis, diabetes, Down syndrome, epilepsy, hemoglobinopathies, other chronic, and relatively rare [32] pediatric diseases (hereafter referred to as ‘other diseases’), and reports of multiple diseases that were eligible for our review. Reports of studies examining diabetes were most common, accounting for one-third (33%) of those included. Articles describing studies of cerebral palsy and epilepsy also comprised substantial proportions of those included (17% and 15% respectively) (Figure 1). Only three reports from the category of ‘other diseases’ focused specifically on children with IEM (two reports examining children with phenylketonuria and one report examining children with maternally inherited mitochondrial disorders and autosomal recessive metabolic disorders) (see Additional file 1). The numbers of children meeting our review’s eligibility criteria were explicitly reported in 283 included reports, with a median number of 76 children (range 6 to 2,101). Studies of diabetes reported the largest median number of children (i.e., 84), while studies of ‘other diseases’ reported the fewest (i.e., 41).

The primary unit of analysis was the child in 43% of 304 included articles; dyadic (caregiver and child) in 28% of reports; the caregiver in 23% of reports; the entire family in 4% of reports, and; a sibling in 1% of reports (Table 2).

**Table 2:** **Report characteristics**

**Disease category**	**# reports**	Median # eligible children studied (range)*	**Primary unit of analysis (#) (% reports) by disease(s)**
Cerebral palsy	51	82.5 (6 - 813)	Child/adolescent (26) (50%)
			Caregiver/parent (9) (18%)
			Dyad - child/caregiver (12) (24%)
			Family (4) (8%)
Cystic fibrosis	11	42 (23 - 136)	Child/adolescent (4) (36%)
			Caregiver/parent (4) (36%)
			Dyad - child/caregiver (3) (27%)
Diabetes	100	84 (10 - 2,101)	Child/adolescent (44) (44%)
			Caregiver/parent (18) (18%)
			Dyad - child/caregiver (35) (35%)
			Sibling (1) (1%)
			Family (2) (2%)
Down syndrome	9	42.5 (25 - 440)	Child/adolescent (1) (11%)
			Caregiver/parent (4) (44%)
			Dyad - child/caregiver (2) (22%)
			Family (2) (22%)
Epilepsy	47	79.5 (9 - 474)	Child/adolescent (26) (55%)
			Caregiver/parent (7) (15%)
			Dyad - child/caregiver (11) (23%)
			Sibling (1) (2%)
			Family (2) (4%)
Hemoglobinopathies	25	59 (7 - 320)	Child/adolescent (8) (32%)
			Caregiver/parent (5) (20%)
			Dyad - child/caregiver (11) (44%)
			Sibling (1) (4%)
Other diseases	29	41 (12 - 272)	Child/adolescent (15) (52%)
			Caregiver/parent (7) (24%)
			Dyad - child/caregiver (6) (21%)
			Family (1) (3%)
Reports of multiple, eligible diseases	32	66.5 (9 - 327)	Child/adolescent (8) (25%)
			Caregiver/parent (16) (50%)
			Dyad - child/caregiver (6) (19%)
			Sibling (1) (3%)
			Family (1) (3%)
All diseases	304	76 (6 - 2,101)	Child/adolescent (132) (43%)
			Caregiver/parent (70) (23%)
			Dyad - child/caregiver (86) (28%)
			Sibling (4) (1%)
			Family (12) (4%)

*Medians and ranges reported on articles for which the number of eligible children was explicitly reported (n = 283) i.e., CP = 50 articles; CF = 10 articles; DM = 94 articles; DS = 8 articles; epilepsy = 44 articles; hemoglobinopathies = 24 articles; other diseases = 27 articles; studies of multiple eligible diseases = 26 articles.

### Patient- and family-oriented outcomes

Across the 304 included articles, we identified 43 unique patient- and family-oriented outcomes within five emergent ‘domains’ or broad categories: general health status and quality of life (3 outcomes); physical health and functional status (11 outcomes); social health and relationships (10 outcomes); mental health (10 outcomes), and; disease management and perceptions (9 outcomes) (Additional file 2). The most commonly measured outcomes were child general health status and quality of life (143 reports, 47%), child mental health (98 reports, 32%), and family function and quality of family life (94 reports, 31%). On the other hand, caregiver cognitive function and the child’s perceived effect of an intervention were reported as having been measured by just one study each.

When reporting on outcomes by disease (Additional file 2), we re-organized the data for 32 articles that incorporated ‘multiple eligible diseases’ i.e., each eligible disease reported in these articles was placed into its respective single-disease category so that each of these 32 articles simultaneously contributed to multiple disease categories (Figure 1). The only disease category for which this resulted in a substantial increase was Down syndrome, almost tripling the total number of included articles reporting on this disease from nine to 26.

### Self-administered measures for common outcomes

We identified 405 independent measures with variable frequency of use across domains, diseases, and outcomes. For readability, we report the top-three most-frequently reported measures for each of the top-ten most frequently-reported outcomes (for those measures appearing in at least 3 articles) (Additional file 1). A complete list of measures by disease and outcome construct is available in an interactive searchable spreadsheet with full references (Additional file 1). Of the top-ten most-frequently-reported outcomes, six were within the domain of mental health (Additional file 3) while none were from the domain of physical health and functional status. Broad constructs such as child general health status and quality of life were measured using a greater number of unique measures (i.e., 74), while more narrow constructs such as child externalizing mental illness were measured using fewer unique measures (i.e., 14) (Additional file 3).

Among the top-ten most-frequently reported outcomes, 28 unique measures were identified (Additional file 3). Dominant measures sometimes emerged for particular outcomes e.g., child externalizing mental illness was reported as having been measured using the Child Behavior Checklist (CBCL) in 31/49 articles reporting on this outcome (63%). Conversely, measures used for other outcomes were more diverse; for example, there were six measures used most frequently for caregiver mental health status, but each one appeared in only three or four of the 64 articles describing this outcome.

Concerning respondents, more than half (54%) of the 28 most-frequently reported measures were reported as offering multiple versions tailored to self-administered response from either caregivers or children (Additional file 3). All but one of the remaining 13 measures were specific to the caregiver (i.e., measures for which child self-report was not relevant).

## Discussion

Our review sought patient- and family-oriented outcomes and their self-administered measures as reported in primary research on children with chronic diseases of relevance to IEM and their families. While other reviews have focused on quality of life in children with chronic illness [33,34], this review is the first to our knowledge that more broadly addresses patient- and family-oriented outcomes and their measures.

Our findings confirm that pediatric chronic disease research into patient- and family-oriented outcomes is relatively well-developed in the field of diabetes [35] as compared with less common diseases such as Down syndrome, hemoglobinopathies [36] and IEM. This likely reflects a larger field of research for diseases with higher prevalence. Most of our included reports focused on the child as the primary unit of analysis, but variation across diseases was apparent. For example, of the 12 reports in our review describing the family as the primary unit of analysis, 4 (33%) were reports of cerebral palsy, with other disease categories contributing 0-2 reports each (Table 2). This difference may be due to chance, and because our search was not exhaustive, it is possible that there is additional literature incorporating family-oriented outcomes that was missed by our search strategy. Nonetheless our findings appear to corroborate acknowledged gaps in family-oriented research, supporting suggestions for further research on this topic [37-39].

The five outcome domains we identified closely parallel those within the PROMIS pediatrics framework [40], although our review additionally describes a domain we labelled ‘disease management and perceptions’. It is possible that this reflects our review’s particular focus on chronic illness for which patient and family perspectives regarding the management of ongoing care are particularly relevant. While only one outcome (i.e., caregiver/child roles in disease management) from this unique domain was among the top-ten most frequently reported outcomes, it is notable that this outcome was most often measured in reports examining diabetes (42/107 (39%)). Diabetes-specific measures also dominated those frequently used to measure this outcome, which may reflect the intensive daily dietary and medical management needs associated with diabetes. While the dietary management of some IEM is relatively more complex, patient- and family-oriented outcomes that have been studied within the field of diabetes are likely to have some applicability to IEM and/or other rare diseases where diet modifications and the importance of metabolic control are relevant.

Of the top 10 most-frequently measured outcomes, six were identified within the domain of mental health. This may reflect our focus on patient/family reports and on self-administered tools in particular, since evidence suggests that results using self-administered measures of mental health might be more valid than those relying on clinician reports [41,42]. However, it could also reflect a tendency of patient-oriented outcomes research in this field to place particular emphasis on mental health as compared with other aspects of the patient and family disease experience [43,44]. Of note, it is unclear whether this emphasis reflects the priorities of patients and families themselves.

Many of the 28 most-frequently reported outcome measures allowed for self-administration by children themselves or by their parents/caregivers, demonstrating respondent versatility. This is important, as parent proxy-reporting of patient-oriented outcomes, such as quality of life, is known to often be discordant with that of children themselves [45]. It appears that, despite long-standing debate around the extent to which children are able to adequately self-report [46], a range of child self-administered outcome measures are available and used within studies of chronic, pediatric diseases requiring ongoing management.

### Strengths and limitations

Our inclusive approach to identifying a range of patient- and family-oriented outcomes and self-administered measures for children with chronic diseases and their families has produced a breadth of findings that is representative of current use in this field of research. We have developed an interactive spreadsheet (see Additional file 1) containing the outcomes and measures that we identified. This tool has potential value for our research in the field of IEM and also for pediatric researchers studying other chronic diseases.

Nonetheless, the scope of this review necessitated methodological tradeoffs that resulted in some limitations. For instance, because we reasoned that outcomes and measures would be used repeatedly across studies, we limited the search to electronic databases. Similarly, our emphasis on outcomes presented challenges when developing the electronic database search strategy because a standardized database lexicon describing outcomes – in particular patient- and/or family-oriented outcomes – is lacking. This made the development of an unbiased, sensitive and specific search strategy particularly difficult. To address this, we relied on the expertise of the working group to identify outcome keywords, and that of an information scientist to implement these according to the review’s objectives. Given the size and scope of the literature of interest, however, eligible studies were certainly missed by our search strategy. Nonetheless, we deemed this limitation acceptable in accordance with our objectives, and acknowledge that we have identified a representative, but not exhaustive, set of articles.

As in other reviews [47], incomplete reporting in our included articles presented additional challenges. For example, the extent to which individual articles described the results of independent studies was often unclear, limiting our ability to report the results of our review with studies as the units of analysis, and rather requiring articles be the unit of analysis. A lack of clarity in reporting also presented challenges for screening and data collection, as it was often not possible to determine whether outcome measures were self- or interviewer-administered. This resulted in the need for an adjudication phase within the screening process and may have resulted in some outcome measures being identified that were not actually self-administered. Finally, while abstracting data, accurate identification of standard outcome measures was challenging as authors used variable naming conventions and referenced different citations, making it difficult to ascertain whether two or more measures were in fact the same. This manifests as a potential limitation on our capacity to definitively identify the frequency with which some measures were reported. These challenges specific to the quality of research reporting represent one of many reasons for developing, implementing and encouraging the use of reporting guidelines [48] to make published research more useful for knowledge syntheses and application [49,50].

## Conclusion

An improved understanding of outcomes that are of primary importance to children and families living with chronic disease requiring ongoing management is critical to informing and supporting patient- and family-centered health care. Our scoping review of the research in this area indicates that currently, there are variable approaches to measuring patient- and family-oriented outcomes. There is an emphasis on mental health outcomes in this literature that may or may not reflect the highest priorities of patients and families themselves. In addition, the comparatively well-developed diabetes literature reports a broad range of patient- and family-oriented outcomes and self-administered measures that may be relevant to diseases, such as IEM, that are more rare.

We suggest that there is a need for expanded study of patient- and family-oriented outcomes within rare, chronic pediatric disease research communities. Such research could build upon the existing literature by incorporating, adapting and validating outcomes and measures that have been well-studied in other disease contexts; and could seek to elucidate additional outcomes that are important to children and their families.

## Additional files

Additional file 1:
**Patient- and Family-Oriented Outcomes and Measures used in Studies of Children/Adolescents with Chronic, Complex Diseases.**
Additional file 2:
**Outcomes by Included Reports and by Disease Category.**
Additional file 3:
**‘Top-three’ measures for top-ten most frequently measured constructs.**

## Abbreviations

CIMDRN: Canadian Inherited Metabolic Diseases Research Network
CIHR: Canadian Institutes of Health Research
IEM: Inborn errors of metabolism
MeSH: Medical Subject Headings
